# A Critical Review of Additive Manufacturing Techniques and Associated Biomaterials Used in Bone Tissue Engineering

**DOI:** 10.3390/polym14102117

**Published:** 2022-05-23

**Authors:** Yanli Wu, Yongtao Lu, Ming Zhao, Sergei Bosiakov, Lei Li

**Affiliations:** 1 Department of Engineering Mechanics, Dalian University of Technology, No. 2 Linggong Road, Dalian 116024, China; wyl0127@mail.dlut.edu.cn (Y.W.); yongtaolu@dlut.edu.cn (Y.L.); zhaoming@dlut.edu.cn (M.Z.); 2 DUT-BSU Joint Institute, Dalian University of Technology, No. 2 Linggong Road, Dalian 116024, China; 3 Faculty of Mechanics and Mathematics, Belarusian State University, No. 4 Nezavisimosti Avenue, 220030 Minsk, Belarus; bosiakov@bsu.by; 4 Department of Vascular Surgery, The Second Affiliated Hospital of Dalian Medical University, No. 467 Zhongshan Road, Dalian 116023, China

**Keywords:** additive manufacturing, materials, bone tissue engineering, biomaterials, polymers

## Abstract

With the ability to fabricate complex structures while meeting individual needs, additive manufacturing (AM) offers unprecedented opportunities for bone tissue engineering in the biomedical field. However, traditional metal implants have many adverse effects due to their poor integration with host tissues, and therefore new material implants with porous structures are gradually being developed that are suitable for clinical medical applications. From the perspectives of additive manufacturing technology and materials, this article discusses a suitable manufacturing process for ideal materials for biological bone tissue engineering. It begins with a review of the methods and applicable materials in existing additive manufacturing technologies and their applications in biomedicine, introducing the advantages and disadvantages of various AM technologies. The properties of materials including metals and polymers, commonly used AM technologies, recent developments, and their applications in bone tissue engineering are discussed in detail and summarized. In addition, the main challenges for different metallic and polymer materials, such as biodegradability, anisotropy, growth factors to promote the osteogenic capacity, and enhancement of mechanical properties are also introduced. Finally, the development prospects for AM technologies and biomaterials in bone tissue engineering are considered.

## 1. Introduction

Additive manufacturing (AM) is a technology that accumulates materials layer by layer, also known as 3D printing. Compared with traditional manufacturing processes, it has significant advantages such as a high degree of freedom in design, low cost, and high efficiency. Since its birth in the 1980s, AM technology has developed rapidly and has had a significant impact on the manufacturing industry, opening up new frontiers for engineering applications in various industrial sectors [1]. Over the past three decades, AM technologies have been widely used in biomedical fields, with advantages such as patient-specific implants. One of the main strengths of this technology is the ability to produce patient-specific devices and treatments. Since there are some mechanisms that are completely material-dependent, AM technology can be considered as a material-based manufacturing technology [2].

It appears that the aging population and the shortage of donor organs [3] have accelerated the demand for bioimplants. Bioimplants are implants used in medical treatment applications, such as porous bone implants. Due to factors such as traumatic injury, birth defects, and cancer, the demand for hard tissue replacement is increasing [4]. Standardized conventional metal implants are currently used to fix and replace these tissue types. However, poor integration of the implants with the host tissues may lead to serious complications such as implant failure and rejection, which will have serious adverse effects on subsequent patient treatment [5]. Therefore, new material implants with porous structures that are suitable for clinical medical applications are gradually being developed. Traditional manufacturing processes cannot produce complex-shaped implants with porous structures. However, AM technologies can offer this possibility. Scaffolds with excellent biological properties can be fabricated using 3D printing technology to provide an ideal microenvironment for cell growth [6], and this has led to the rapid development of AM technologies for biomedical applications such as teeth, bones, and spinal cords.

Bone tissue engineering (BTE) aims to design a bone implant as a bone substitute to achieve the purpose of bone repair and tissue regeneration. To achieve this goal, a variety of materials have been used to develop biological structures that can replace human bone. Materials for bone tissue engineering must possess biocompatibility and suitable mechanical properties that meet the requirements. Such materials include natural and synthetic polymers, ceramics, and metals. Different materials require different processing methods due to their characteristics, and their medical applications are also different. However, a basic requirement is the need for them to be tested for safety and compatibility with human tissue. Complex and unique processed parts can be obtained through AM technology realization [7]. Porous metallic bone implant materials with good biocompatibility and mechanical properties are widely used. However, the existing materials inevitably have many problems such as anisotropy and biodegradability. Therefore, polymer materials with high biocompatibility, excellent biodegradability, and suitable non-toxicity are used to produce scaffolds for bone tissue engineering. However, polymers have poor mechanical properties compared with metals, and therefore they are often combined with other materials to improve the performance. Accordingly, by studying the properties and processing methods of different materials, it is possible to prepare bone implants that are non-toxic to humans, can promote bone formation, and are closer to human bone in all respects.

This paper intends to provide a critical review of different AM technologies and the recent progress in the use of various materials including metals, natural polymers, and synthetic polymers that are suitable for AM technology in biological tissue engineering. First, the advantages of several AM technologies, the applied materials, and the corresponding recent advances in biomedicine are introduced. Then, the characteristics of metal biomaterials and polymer materials, as well as their commonly used AM technologies and related progress are summarized. The research progress in materials for tissue engineering, especially those related to bone, is highlighted. Finally, this review concludes with a discussion of the existing challenges from the perspective of AM technologies and materials, while presenting future opportunities and prospects. 

## 2. Additive Manufacturing Technologies

**Figure 1:**
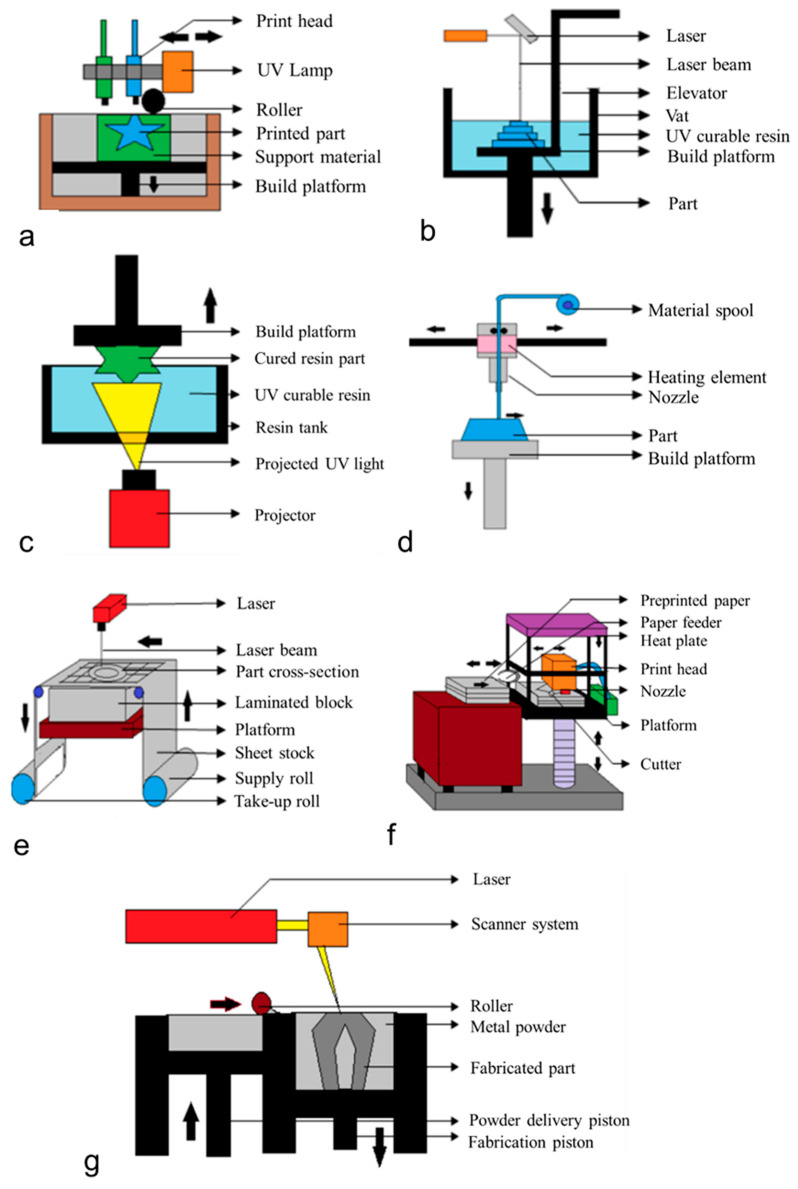
Schematic diagram of popular additive manufacturing processes: (**a**) PolyJet printing; (**b**) stereolithography (SLA); (**c**) direct light processing (DLP); (**d**) fused deposition modeling (FDM); (**e**) laminated object manufacturing (LOM); (**f**) selective deposition modeling (SDM); (**g**) selective laser sintering (SLS) [1].

The American Society for Testing and Materials (ASTM F2792-12a) has developed a set of standards that divides the range of additive manufacturing processes into seven broad categories [8]. According to ASTM, AM processes can be classified, based on the method of forming the final components, into the following seven types: (1) material jetting (MJ), (2) binder jetting (BJ), (3) vat photopolymerization (VP), (4) powder bed fusion (PBF), (5) material extrusion (ME), (6) energy deposition (DED), and (7) sheet lamination (SL) [1]. Figure 1 shows typical processes for the above-mentioned categories. Table 1 summarizes the methodology, commonly used processes, advantages, limitations, and commonly used materials for tissue engineering using the above AM processes. The properties of the fabricated parts depend not only on the starting materials used but also on the characteristics of the AM process [9,10]. Therefore, in the following sections, the advantages and disadvantages of several widely applicable AM technologies and suitable materials will be introduced, providing new ideas for related research on improving the performance of the parts. In addition, recent research progress in existing AM processes in the biomedical field is also introduced.

### 2.1. Material Jetting (MJ)

Material jetting (MJ) is one of the fastest and most accurate 3D printing technologies. It works by using liquid materials deposited in different ways onto the build platform and then cured by means of photopolymerization to create 3D parts. Since it uses multiple print heads, it enables multi-material printing, which is its key advantage [1]. MJ is able to create parts with very smooth surfaces that even match the appearance of injection-molded parts. At the same time, MJ also has some limitations, including high cost (it is one of the most expensive 3D printing technologies) and poor mechanical properties. Materials commonly used in the process include photosensitive resins, thermoplastics, waxes, reactive materials, etc. [19].

The representative technology is the PolyJet technology developed by Stratasys. This is relatively new in the field of additive manufacturing, and it can ensure that multiple materials are sprayed and cured at the same time to achieve multi-material printing. A common material used is resin, but other photopolymers are also used for 3D printing for dentistry, medical, and biomedical applications. Kitamori et al. [20] developed a mouthpiece made of the biocompatible class VI resin PolyJet photopolymer Object MED610, based on PolyJet technology, as a fixation device for patients undergoing head and neck radiation therapy. Danilo et al. [21] analyzed the ability of the Polyjet model to reproduce the mandibular anatomy and its dimensional errors and found that the technology could accurately reproduce anatomical details. Rasheed et al. [22] utilized PolyJet technology to study the fabrication of bone scaffolds. Research has shown that the PolyJet printing technology can be used to print 3D scaffold structures with restricted porosity levels, thereby providing permeability in a range similar to that of human bone.

### 2.2. Binder Jetting (BJ)

Binder jetting (BJ) is a powder-based AM process in which an inkjet printhead is used to selectively eject a liquid binder that binds powder particles in a powder bed together by deposition. Because the printing process does not involve high temperatures and does not require a laser, it enables the manufacture of large parts at a low cost. Without a supporting structure, it enables complex parts to be made from strong materials [19]. However, the mechanical properties of the parts produced by the BJ process are poor, as there is no process such as sintering or melting. Therefore, subsequent steps such as infiltration or sintering are necessary to strengthen the structure of the parts [23], which makes the entire process longer and more expensive. The BJ process can print a variety of powder materials including ceramics, metals, sand, and polymers [24]. In the biomedical field, it is often used to fabricate bone tissue engineering (BTE) scaffolds. Non-metallic biomaterials such as hydroxyapatite (HA), calcium phosphate, and calcium silicate can all be used in production.

### 2.3. Vat Photopolymerization (VP)

Vat photopolymerization (VP) is one of the first additive manufacturing processes utilizing photosensitive materials to selectively harden photosensitive liquids into 3D objects under suitable laser irradiation. It can manufacture parts with complex geometries, as well as create concept models and rapid prototyping [1]. The VP process can be further divided into stereolithography (SLA), digital light processing (DLP), and volume 3D printing.

One of the most representative processes is stereolithography (SLA). This directly generates prototypes from CAD data, and therefore it has the advantages of fast processing speed, a short production cycle, and no need for secondary processing. It uses a wide range of printing materials, and the surface quality and precision of the manufactured parts are high. However, it also has the following limitations: (i) high cost, (ii) high environmental requirements, and (iii) complicated operation. It is suitable for processing photopolymers and resins but is limited to processing materials that polymerize under light. Due to the development of high-precision SLA 3D printers and the optical, mechanical, and thermal properties of novel resin formulations, the SLA process offers opportunities in numerous fields including engineering, dentistry, and medicine. Zhou et al. [25] developed 3D printing of a beta-TCP green body using SLA technology, to repair hard tissue. After adding an optimized KH-560 dispersant, the subsequent SLA 3D printing material had good fluidity and stability, and uniform dispersion. Burke et al. [26] demonstrated that SLA-prepared hydrogels have similar thermal and chemical properties to the hydrogels prepared by ultraviolet (UV) cavity photopolymerization. However, the former have higher compressive strength and tensile stiffness, and better hydrophilicity, and can therefore be more suitable for BTE applications.

Digital light processing (DLP) utilizes digital microscope equipment to project a photomask to cure each layer separately in a short period of time. It prints faster than SLA, but it is inherently less accurate and requires post-processing. The process enables the processing of different biomaterials, including polymers and their composites, for biomedical applications [27]. In order to solve the problem of its low precision, researchers have carried out a series of studies. Li et al. [28] developed an analytical model based on differential analysis to obtain the relationship between the UV exposure time and the cured thickness of the monolayer. In this model, the analytical Jacobs working curve can be described as depending only on three properties of photocurable materials: solid absorbance, liquid absorbance, and gelation time. The analytical Jacobs working curve used polyethylene (glycol) diacrylate (PEGDA) hydrogel and gelatin methacrylate (GelMA)/decellularized extracellular matrix (dECM) bio-ink to predict the DLP printing parameters, which were found to be sufficient for accurate printing of 3D complex structures. This verification provides the possibility for high-precision DLP printing of photocurable materials.

### 2.4. Powder Bed Fusion (PBF)

Powder bed fusion (PBF) is one of the most widely used AM processes and is the process used for the preparation of most metal implants. In this process, a laser or other radiation is used to selectively melt powder particles, which are then printed layer by layer with a replica knife to build a 3D object. The PBF process has several advantages, including: (i) flexible design, (ii) good resolution, and (iii) less material waste, and the powder left in the process can be recycled and reused. At the same time, some of its main limitations are: (i) long printing time and (ii) the need for post-processing to improve the mechanical properties, such as reducing residual stress, reducing surface roughness, etc. Since it can theoretically handle any material in powder form, its range of materials is broad and includes polymers, metals, alloys, and ceramics [29]. Common processes for PBF include: (i) selective laser melting (SLM), (ii) selective laser sintering (SLS), and (iii) electron beam melting (EBM).

In SLM, lasers are commonly used to provide the energy to melt metal powders, and the process is commonly used for biomedical metal materials such as 316L stainless steel, cobalt–chromium alloys, and titanium (Ti) alloys [30]. Metal parts with compact structures, high dimensional accuracy, and good mechanical properties can be directly manufactured by this method. In their latest study, Tonelli et al. [31] achieved the melting of Co–28Cr–6Mo samples using SLM technology. They determined the optimal LED window for Co–28Cr–6Mo alloys for biomedical applications and maximized the overall quality of the SLM parts. In addition to the above metals, Shishkovsky et al. [32] investigated the process conditions for SLM fabrication of nickel–titanium alloy parts and found that the relative bulk density of the nickel–titanium alloy after laser cooling was about 97% of that in the solid state, proving that the alloy could be used for bone scaffolds.

In SLS, a laser beam is used to sinter a polymer material to form a layer, and these layers are continuously built up to form a 3D object. Commonly used materials for SLS are mainly polymers, but also include ceramics. The advantage of this method is that it can be applied to any powdered biomaterial that is not decomposed under the laser beam, to prepare scaffolds [33], including materials such as polyetheretherketone (PEEK) and hydroxyapatite (HA) [34,35]. Tan et al. [34] chose an SLS RP system to perform experiments on the above biomaterials to verify biocompatibility, since SLS technology can achieve good control over the microstructure of the fabricated scaffolds by adjusting the process parameters. This process is suitable for printing biocompatible materials to fabricate porous bone implant scaffolds, and it has the ability to change the macrostructure of scaffolds by changing the basic cellular design of the scaffolds to form scaffolds with different pore sizes. The biocompatibility of the scaffolds printed by this process has been experimentally verified [36]. Therefore, it is used to print scaffolds for cardiac and skeletal muscle tissue engineering. For example, it can utilize tissue scaffold computer-assisted (cast) systems to create low-stiffness porous PCL scaffolds suitable for the heart [27].

Electron beam melting (EBM) works in a similar way to the SLM technology described above, except that a high-energy electron beam is used instead of a laser to melt the metal powder particles. It can process various types of metal biomaterials such as 316L stainless steel, titanium alloys, and cobalt-based superalloys. The most commonly used material is a titanium-based alloy [37], and this is also one of the most commonly used metals for bone implants. Aziziderouei et al. [38] investigated how the Charpy impact energy (IE) of Ti–6Al–4V is affected by the build direction and the lack of fusion (LOF) during the EBM process, and found three influencing factors related to the notch direction (ND) as well as the direction of crack propagation in the microstructure: BD (which means that IE increases with an increase in the angle between the construction direction and the notch direction), LOF, and the difference in the microstructure. Both SLM and EBM can be used to process metallic materials, and they work in similar ways. However, the process used to print porous bone implant materials such as titanium (Ti) alloys in bone tissue engineering is usually the SLM process. In addition, the SLM process can also be used to process degradable materials such as magnesium (Mg) and iron (Fe). Compared with SLM, there are fewer applications for EBM. Regarding biomedical metals, the EBM process is used to process tantalum (Ta). SLS is different from the above two methods in that its printing material is a polymer. It is the most common processing method for processing polymers and it can print synthetic polymers such as PLA and PCL.

### 2.5. Material Extrusion (ME)

Material extrusion (ME) is one of the most widely used 3D printing technologies in both industry and households. It is also known as fused deposition modeling (FDM) or fused filament fabrication (FFF). It selectively extrudes a spool of material filaments through a nozzle and builds a 3D object layer by layer. Its main advantages are: (i) less pollution as there is no solvent removal, (ii) a variety of material options, and (iii) lower cost. It is one of the most affordable AM processes. However, its main limitation is anisotropy, resulting in poor mechanical properties and high porosity in the molded parts. The process can extrude a variety of materials such as commonly used thermoplastics, as well as high-performance biomaterials such as polylactic acid (PLA) and PEEK [39].

Unlike FDM, direct ink writing (DIW) is an ME process that can print relatively viscous, concentrated polymer solutions. It is also known as 3D dispensing or 3D drawing. Its strength lies in its variety of material options, and it is suitable for the widest range of printable materials available. Due to the diversity of its machinable materials, the nozzle size of DIW varies [19]. Recently, Skylar-Scott et al. [39] developed a DIW-based multi-material multi-nozzle 3D printing system that offers the possibility of fabricating soft matter with high material complexity and a high build rate. In recent years, there has also been progress in liquid deposition modeling (LDM) [40], which uses ink materials.

### 2.6. Directed Energy Deposition (DED)

Directed energy deposition (DED), also known as laser metal deposition (LMD) or laser energy net shaping (LENS), is a relatively complex 3D printing process that uses different light sources such as lasers, electron beams, and plasmas to melt materials, which are then selectively deposited in layers that are built up layer by layer to form 3D objects. Compared with other processes such as PBF [41], it can easily switch materials to realize multi-material printing. It can improve the mechanical properties and has higher cost-effectiveness. The main limitations of DED include: (i) limited complexity, (ii) deformation due to temperature changes, (iii) higher surface roughness, and (iv) the need for post-processing [41] to remove residual stress.

The process uses a wide range of materials, including metal powders such as tantalum, titanium, stainless steel, aluminum, and cobalt [5]. It uses less polymer, and the injected material is required to be a powder or filament. Its development is more advanced than the following two processes that can be deposited at the same time as material extrusion and powder bed melting. In addition, it has proven capabilities for the fabrication of porous biomedical implants [42] and functionally graded materials [43]. However, according to a survey, most of the porous implants currently used in biomedicine are produced by PBF technology, and only a few are produced via DED technology. The main reason may be related to production, since the low precision produced by DED limits the minimum size and properties.

### 2.7. Sheet Lamination

Sheet Lamination (SL) is a technology that bonds and stacks sheet materials layer by layer to build 3D objects. Compared with other processes, it has low cost and can be used to manufacture large parts quickly. Laminated object manufacturing (LOM) is one of its representative processes, in addition to which other concentrated processes have also been developed. Materials used in the LOM process include sheet metal, ceramics, composite fibers, thermoplastic foils, etc. However, it is too difficult to manufacture internal structure during the cutting process, resulting in the waste of materials, and therefore this method has not been widely used.

In summary, among the above AM processes, the processes commonly used for polymers include MJ, BJ, VP, ME, and SLS, and the processes commonly used for metals include SLM, EBM, and DED. At present, the technologies widely used in bone tissue engineering (BTE) are PBF, SLA, and BJ. For the PBF process, which is the most widely used technology, the most commonly used material for SLM and EBM is metal, and the most commonly used material for SLS is polymer, which has been widely used in BTE to process scaffolds. In addition, the BJ process is able to fabricate BTE scaffolds. Processes that have achieved multi-material printing include MJ, DIW, and DED, providing the possibility of the creation of new AM technologies.

## 3. Metallic Biomaterials for AM

Metals are widely used in AM technology due to their good mechanical properties and processability. At the same time, some metals are used in biomedicine, mostly in bone implants and tissue engineering, due to their good biocompatibility. Furthermore, the use of AM process printing can meet the individual needs of patients [44]. Various types of metals have different processing methods and applications due to their different properties [45]. Table 2 summarizes several commonly used metal biomaterials in AM and their advantages, limitations, and applications in bone tissue engineering. The current research progress for each material is also discussed in the following subsections.

### 3.1. Ti-Based Biomaterials

Titanium (Ti) and its alloys have good biocompatibility and corrosion resistance due to the stability of the titanium oxide (TiO_2_) layer on the surface. Compared with other metals, Ti has excellent mechanical properties, making it widely used in bone implants. It has become an important material for the replacement of human hard tissue. However, due to the poor tribological properties of Ti-based implants and their susceptibility to environmental influences, Ti alloys need to be developed and improved to meet the needs of BTE. For example, surface modification of titanium alloys is carried out by methods such as micro-arc oxidation (MAO), ion implantation, plasma spraying, laser surface modification, sol–gel technology, friction stir processing (FSP), etc. [50].

**Figure 2:**
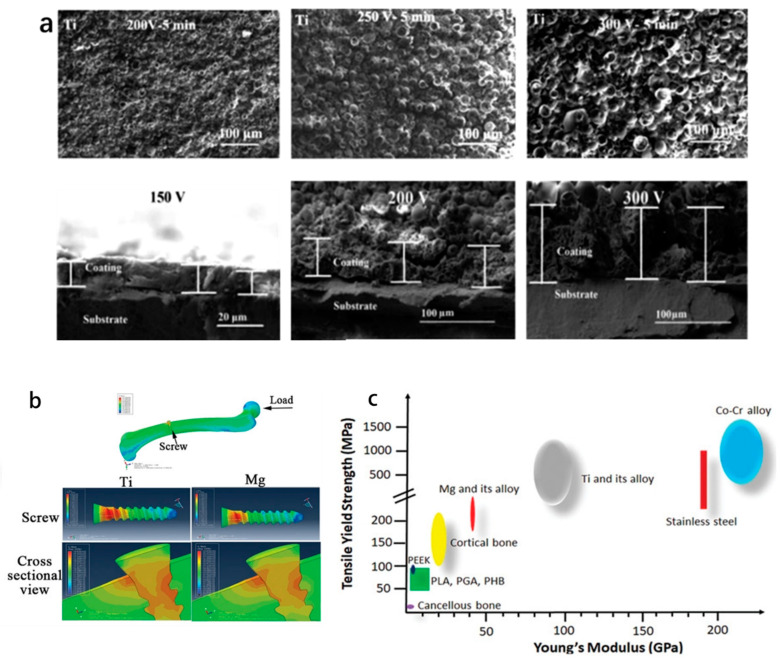
Research related to Ti: (**a**) SEM micrographs of wollastonite calcium phosphate (W-CaP) coatings on Ti surface, and SEM micrographs of cross-sectional W-CaP coatings produced on Ti at different voltages [64]; (**b**) plot of peak stress distribution in a finite element analysis model consisting of the femoral head and the inserted titanium or magnesium screw, where the data surface has a lower stress distribution in the bone tissue around the titanium screw compared to the magnesium-based screw and the stress at the surface titanium-implant–bone interface shield [48]; (**c**) tensile yield strength distribution range of each biomaterial [48].

Ti alloys are excellent bone replacement materials due to their light weight, high density, high mechanical strength, and corrosion resistance. A bone substitute material must have good biocompatibility and excellent mechanical properties [51]. The above requirements can be achieved using AM technology to manufacture bone implants with porous structures whose stiffness can be close to that of natural bone [52]. Ti–6Al–4V is an (α + β)-type alloy which has been widely used in BTE and is a well-known medical-grade titanium alloy. After heat treatment, the mechanical strength of Ti–6Al–4V can be increased by 50% without obvious influence on the Young’s modulus [53]. Therefore, it can be used in load-bearing applications such as fracture fixation plates [54]. However, as a permanent implant, it is cytotoxic due to its Al and V content [55]. Therefore, aluminum-free and vanadium-free Ti alloys have been introduced into implant applications [56]. In recent years, numerous studies have been devoted to exploring the ability of titanium surface modifications and coatings to reduce bacterial adhesion and inhibit infection. The following figure shows the research results related to Ti and its alloys (Figure 2). Hengel et al. [57] employed the SLM process to fabricate porous titanium implants with interconnected pores, and they achieved biofunctionalization by embedding silver nanoparticles in the surface layer of the oxide grown using plasma electrolytic oxidation (PEO) in a Ca/P-based electrolyte. The test showed that the implant was non-cytotoxic and had strong antibacterial activity. 

The commonly used metal AM process for Ti alloys is LPBF, and the samples produced by this method have a relatively high strength as a result of the change in the microstructure. However, it is inevitable that during the melting process, anisotropy will be caused in the material [58,59,60]. Lu et al. [61] took Ti–6.5Al2Zr–1Mo–1V as a research object, adding 0.1 wt% of boron. The coarse columnar b grains and the continuous grain-boundary a phase were effectively refined, thereby reducing the mechanical anisotropy. Yao et al. [62] constructed a layered structure by alternately depositing Ti–6Al–4V layers and boron-modified Ti–6Al–4V layers, based on two-line electron beam directional energy deposition (EBDED). This was proved to be capable of effectively reducing anisotropy, providing a prospect for further broadening the application scope of additively manufactured titanium alloys. At present, the osseointegration of titanium implants is a problem that remains to be solved. Molina et al. [63] immobilized covalent dendrimers on titanium implants, synthesized amide-based amino-terminal dendrites, and coupled them to the titanium surface in a controllable manner. The dendritic portion anchored to the implant surface provides a scaffold for extracellular matrix (ECM) protein components that facilitate cell adhesion and proliferation.

### 3.2. Ta-Based Biomaterials

Tantalum (Ta) has good corrosion resistance, mechanical stability, and mechanical properties, and it has better biocompatibility and fatigue resistance than traditional metal materials such as titanium, magnesium, and various alloys. Its surface can form a layer of bone-like apatite, which can be strongly combined with bone and can enhance bone ingrowth [65], and therefore it has received more and more attention in bone tissue engineering.

**Figure 3:**
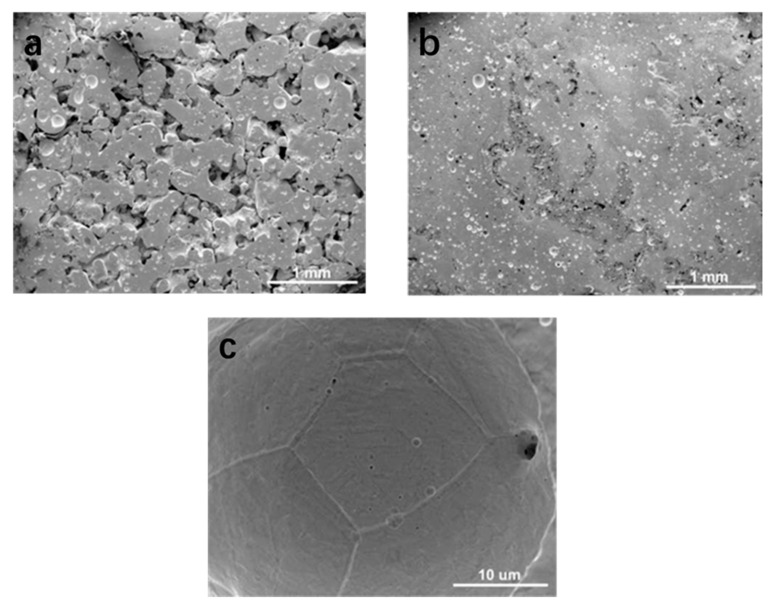
Microstructural study of Ta: (**a**) FESEM microstructure of porous tantalum structures; (**b**) porosity characteristics of 55% and 73% dense samples; (**c**) Lens Grain™-processed porous Ta [66].

The elastic moduli of pure tantalum and bone tissue are quite different, which is not conducive to osseointegration. Bella et al. [66] utilized laser engineered net shaping (LENS) to process Ta to construct porous Ta implants with various relative densities (45%–73%), yield strengths (100–746 MPa), and Young’s moduli (2–20 GPa). The figure below shows an FESEM microstructure study of the porous Ta structure (Figure 3). As a new type of bone implant, porous Ta has the characteristics of high porosity and low elastic modulus, and its internal structure is interconnected. Due to the structural characteristics, its elastic modulus is between those of cortical bone and cancellous bone, and therefore it is suitable for arthrodesis, joint replacement, and cartilage repair [67]. Wang et al. [68] designed porous Ta and Ti implants with the same pores using CAD and fabricated them using SLM to demonstrate that the porous Ta has comparable biological performance to traditional porous Ti implants for small bone defect repair. 

Due to its high density, high cost, and high melting point, the processing of porous Ta is more challenging than that of other metals [68]. However, it can be produced by the AM process, using an optimized design and tuning the parameters, etc., to adjust its mechanical properties. This can also be achieved by adding other metal powders. Tang et al. [69] fabricated dense- and fine-lattice tantalum structures using the electron beam melting (SEBM) process, and the printed Ta (99.90%) achieved a tensile ductility of 45%, far exceeding the minimum tensile requirement. In addition, hybrid titanium–tantalum alloys such as the Ti–50Ta structure [70] have been developed, with excellent biocompatibility and corrosion resistance. Therefore, Ta alloys have received extensive attention in the past decade and have been used in medical applications such as femoral head necrosis treatment and coronary stent implantation.

### 3.3. Mg-Based Biomaterials

Magnesium (Mg) and its alloys exhibit natural degradability in addition to their good mechanical properties and biocompatibility. As a representative of biodegradable materials, due to its biomechanical properties and in vivo degradation characteristics similar to natural bone tissue, Mg has obvious advantages in BTE. Its density and elastic modulus are closest to those of human bone tissue, and it has similar mechanical properties to bone, which can reduce stress shielding to a certain extent [71]. Magnesium, the fourth most common mineral in the human body, participates in a variety of biological reactions and can be absorbed by the digestive system. It also has the function of promoting bone growth [72]. The release of magnesium ions can stimulate the formation of bone and accelerate healing.

Mg is defined as a “biodegradable metal”. After implantation, it can be gradually degraded and absorbed, eventually being replaced by newly formed tissue, [73]. However, the extremely high degradation rate of Mg results in the release of hydrogen. Therefore, extensive research has been carried out to control its degradation rate. Alloying magnesium by adding rare earth elements (Y, Nd, Gd, etc.) to Mg to form alloys [2] is a common way to reduce its degradation rate, and alloying Mg with manganese (Mn) can improve its corrosion resistance [74]. In addition, Mg–copper or Mg–zinc can improve the strength of Mg [53]. The Mg–Nd–Zn–Zr alloy (JDBM) developed by Wang et al. [75] has good corrosion resistance and antibacterial properties, and can be used in mandibular fracture screws and cardiovascular stents. Surface modification treatments and coatings can also reduce magnesium degradation rates. Studies have shown that Ca–P [76,77], as a magnesium alloy coating, can effectively reduce the degradation rate and prevent mechanical loss. Kopp et al. [78] utilized heat treatment and plasma electrolytic oxidation (PEO) to modify the structures of magnesium scaffolds with different pore sizes prepared with LPBF. The results demonstrated that the long-term stability of the small-pore PEO-modified scaffolds was improved, while the degradation performance of the scaffolds after heat treatment declined. 

The AM preparation of Mg powder is more challenging due to its characteristics. In recent years, the SLM process has been used for the preparation of biodegradable magnesium [79]. In the field of biomedicine, Mg is mostly used in bone implant materials (bone screws [48]) and vascular stents [80]. In China, the Mg-based bone fixation implants being currently developed are 99.99% high-purity magnesium [81], and pure Mg screws have been officially approved for the treatment of steroid osteonecrosis in the China Medical Product Management Multicenter Clinical Trial [48]. The development of Mg has very broad prospects in the field of bone tissue engineering.

### 3.4. Fe-Based Biomaterials

Iron (Fe) and its alloys are used as potential biodegradable materials for biomedical implants [82]. Compared with other metal implants, Fe-based materials have better ductility and mechanical strength, and their yield strength and elastic modulus are high. In addition, Fe has good mechanical properties and biocompatibility. Therefore, it can be used as a weight-bearing implant and a bone overload replacement. Iron is one of the essential trace elements for the human body, but it is toxic at high concentrations [83,84,85]. Therefore, Fe-based implants with porous structures have been extensively investigated. Various preparation methods are used for Fe scaffolds with various pore sizes [86], and Montani et al. [87] reviewed the preparation of biodegradable Fe materials using SLM.

However, pure Fe implants degrade slowly in the physiological environment. In order to accelerate the degradation rate, other elements are often alloyed with iron. Carluccio et al. [88] found that the corrosion rate of prepared Fe–Mn porous scaffolds with good biocompatibility and cell activity was much higher than that of pure iron scaffolds. Apart from Mn, the addition of other noble metals such as Pt, Pd, Au, and Ag [89,90] can lead to the formation of Fe-based secondary phases, which can promote iron biodegradation and accelerate the degradation rate without other negative effects. In addition to the degradation rate, the improvement of the mechanical properties of Fe-based materials is also a research focus. By preparing porous Fe with different porosities, Čapek et al. [91] found that, with an increase in porosity, the mechanical properties and average hardness of Fe decreased, with the former effect being more obvious.

Compared with Mg-based biomaterials, Fe-based materials have the advantage of not releasing hydrogen. However, the degradation and absorption properties of Fe-based implants are not yet clear [82], and therefore they have not yet entered the clinical stage, as further research is needed.

### 3.5. Other Metallic Biomaterials

**Figure 4:**
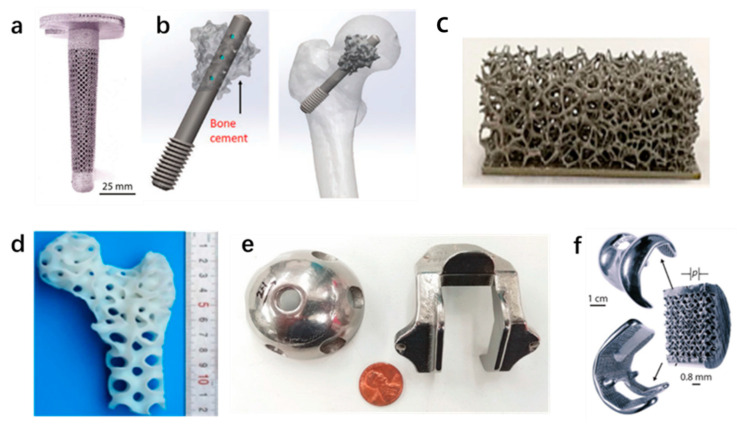
Examples of the application of various metals in biological tissue engineering: (**a**) Ti-64 tibial shaft structure [94]; (**b**) magnesium-based screw with holes in the shaft for injection of bone cement to repair femoral head necrosis [48]; (**c**) Magnesium porous bone scaffold [72]; (**d**) magnesium bone implants using laser additive manufacturing [72]; (**e**) 316L stainless steel manufactured by PBF process [95]; (**f**) Co–Cr knee implant [94].

In addition to the metallic biomaterials mentioned above, other metallic biomaterials such as cobalt, chromium, and zinc are also included. Figure 4 shows examples of the application of various metals in BTE. Due to their superior hardness, good wear resistance, and corrosion resistance, cobalt–chromium alloys are widely used in dental implants [92]. As well as common metals, smart alloys (also known as shape memory alloys) are currently receiving widespread attention, because they can remember and restore their original shape when subjected to external stimuli or reactions with other chemicals. The most famous shape memory alloy for biomedical applications is Nitinol [93]. Various AM technologies such as PBF have been applied for processing this material, and its further development may lead to the four-dimensional (4D) printing of biomedical implants, which is a future trend.

In summary, as mainstream bone implant materials, metals are the focus of current research. Among them, Ti alloy is the most widely used bone implant material, commonly processed using SLM and DED. Furthermore, the most commonly used process for bone scaffolds in BTE is the SLM process. Due to the low precision of DED technology, the minimum size of the structure is limited to a certain extent, resulting in its limited use in medical applications. In addition to non-degradable alloy materials such as titanium and tantalum, degradable alloys such as magnesium (Mg) and iron (Fe) have received extensive attention in biomedicine. Mg is often used for bone screws and vascular scaffolds for bone tissue engineering. Regarding degradable materials, current research directions include methods of controlling their degradation rate (such as alloying, surface modification, etc.) and the impact of biodegradation and absorption on the in vivo environment. The above alloys are difficult to process due to their material properties, and therefore the current commonly used 3D printing process is SLM. In addition, processes such as EBM and DED can also be used for the processing of titanium, tantalum alloys, etc.

## 4. Polymer Materials for AM

**Figure 5:**
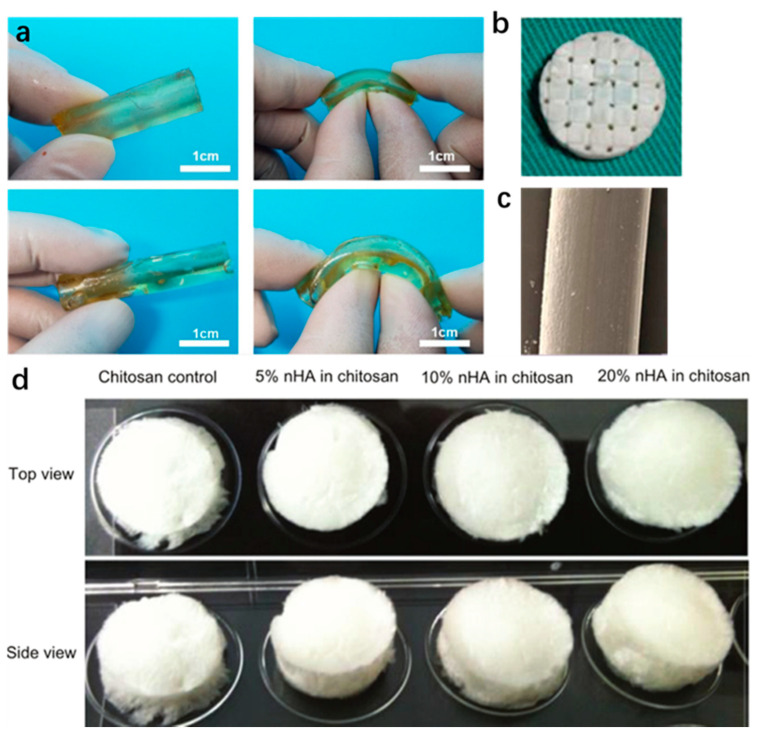
Applications of common polymers in biological tissue engineering: (**a**) morphology of 20peg-1.0C/H and 40peg-1.5C/H pipes [96]; (**b**) silk fibroin scaffold [97]; (**c**) PLA bone implant [98] (the figure can be viewed at wileyonlinelibrary.com); (**d**) chitosan/nHA scaffold prepared after freeze drying [99] (From International Journal of NanoMedicine 2012 7 2087-2099’ Originally published by and used with permission from Dove Medical Press Ltd.).

In addition to metals and ceramics, biomaterials also contain polymers. These can be divided into natural polymers (such as collagen, gelatin, silk fibroin [SF], chitosan, hyaluronic acid [HA], etc.), synthetic polymers (such as polylactic acid [PLA], polyurethane [PU], polyethylene glycol [PEG], polycaprolactone [PCL], etc.), and their composites. Figure 5 shows applications of commonly used polymers in BTE. Table 3 summarizes several commonly used polymers along with their advantages, limitations, and applications in biomedicine. The following sections discuss these in detail.

### 4.1. Natural Biopolymers

Natural polymers contain trace amounts of functional structural molecules that can promote cell growth. Natural polymers include collagen, gelatin, chitosan, silk fibroin (SF), hyaluronic acid (HA), etc.

Silk is a natural biopolymer composed of protein, silk fibroin, and sericin, and silk fibroin (SF) is obtained after degumming. Due to its good biocompatibility, excellent processing properties, and controllable biodegradability, SF is often used as a biodegradable material in BTE, for example, in silk fibroin scaffolds. SF scaffolds can be processed by 3D printing [97]. The use of AM processes (such as 3D printing) can better control the scaffold structure, which will affect the mechanical properties of the scaffold to a certain degree. SF has received attention due to its unique properties. Cheng et al. [106] utilized an SF coating to load vascular endothelial growth factor (VEGF) onto the surface of polymer-based bone implants and found that it could significantly improve graft healing. Mazaher et al. [107] developed a three-dimensional (3D) bilayer scaffold made of biologically decellularized human amniotic membrane (AM) with viscoelastic electrospun nanofibrous silk fibroin (ESF) spun on top and investigated its biomechanical properties. Compared with AM alone, AM/ESF exhibited significantly improved mechanical properties and superior cell adhesion and proliferation. Choi et al. [108] utilized indirect 3D bioprinting technology to prepare silk fibroin scaffolds for use in bone tissue engineering and found that by changing the concentration of silk fibroin and the solvent in the silk fibroin solution, the mechanical strength and flexibility of the scaffolds, respectively, could be adjusted. However, data on the short-term and long-term effects of SF degradation products in vivo are currently unclear. Therefore, further exploration of host responses to SF is required.

Chitosan is a natural polymer composed of d-glucosamine and N-acetylglucosamine, which comes in various forms such as beads, membranes, and hydrogels. Chitosan is not only biocompatible and degradable but also has antibacterial, antifungal, and antitumor activities. In addition, it is non-toxic to tissue, and thus it is applicable in BTE [101,109]. However, due to its poor mechanical properties, it is often mixed with other materials to form more ideal materials [110,111,112]. Wattanutchariya et al. [113] fabricated scaffolds with open-pore structures using a mixture of chitosan, gelatin, and hydroxyapatite (HA). Arumugam et al. [114] synthesized biocompatible carbon nanotube nanohybrids using chitosan, polyacrylamide (PAM), and polylactic acid. Owen et al. [99] prepared three-dimensional porous chitosan scaffolds with different concentrations of nanocrystalline hydroxyapatite and SWCNTs, which are beneficial for bone regeneration. After 10 min of sonication, they were homogenized, distributed in molds, frozen at −80 °C, and lyophilized. Figure 5d shows the obtained 3D porous scaffold. He et al. [115] prepared a hybrid bio-ink made of photocurable chitosan and acrylamide, which can be used for digital light processing (DLP)-based 3D printing. The DLP-based 3D printing process is suitable for processing complex 3D hydrogel structures with high strength and good biocompatibility.

Collagen is widely used in BTE due to its excellent biodegradability, biocompatibility, and immunogenicity, as well as its ability to form tissues and grow cells. Type I collagen is most common in bone tissue and is used as a scaffold material in BTE [116]. Although type I collagen has good biocompatibility and osteoconductivity, it also has some limitations, including high biodegradability, low mechanical strength, and lack of osteoinductive activity. Accordingly, numerous attempts have been made to improve type I collagen-based implants for BTE. Sun et al. [117] utilized collagen powder as a scaffold for the administration of human umbilical-cord-derived mesenchymal stem cells (HUC-MSCs) in a rabbit model of alveolar fissure. They evaluated the effect of collagen granules combined with HUC-MSCs on the repair of alveolar cleft bone defects and found that collagen particles combined with HUC-MSCs were significantly better than collagen particles alone in inducing bone repair and regeneration. Klüver et al. [118] first successfully printed a BioScaffolder 3.2 from GeSiM mbH via the AM process using collagen, water, and glycerol. The cytoplasmic matrix (ECM) plays an important role in the structural support of tissues, and proteins extracted from the ECM have been used to fabricate scaffolds. Obregon-Miano et al. [119] utilized a porcine bone demineralized and digested extracellular matrix (pddECM) containing collagen type I mixed with 20% w/v polyethylene glycol diacrylate (PEGDA) to create a polymer grid bracket. Compared with natural polymers, the polymer scaffolds had structural stability and showed rapid degradation. As major organic components of bone tissue, collagen-based biomaterials may become one of the most commonly used materials in biomedical applications, especially BTE [120].

### 4.2. Synthetic Polymers

The types of polymers most used in bone tissue engineering include polylactic acid (PLA), polyglycolic acid, polyethylene glycol (PEG), polycaprolactone (PCL), etc.

Polylactic acid (PLA) is a thermoplastic polyester with good biocompatibility, excellent mechanical and thermal properties, and good processability, which is usually processed and studied using AM. As one of the polymers processed using FDM, PLA is widely used in biomedical implants as a biodegradable material [121]. However, it suffers from limitations such as slow degradation rate and low impact toughness, and therefore blending PLA with other polymers to improve related properties and generating new PLA polymers/blends for targeted applications have been considered. Hydroxyapatite (HA) has been proved to effectively enhance the mechanical strength of PLA, and it has been verified that composites with an HA content of 30% exhibit the highest compressive, flexural, and impact strengths [122]. Wu et al. [123] utilized fused deposition modeling (FDM) technology with a composite of degradable polymers, i.e., PLA and HA as a filament, to evaluate three types of PLA/HA composite formulations containing 5-10-15 wt% of HA. They considered that the addition of HA to PLA can improve the mechanical properties of 3D printed models. Vidakis et al. [124] employed the FFF process using silica (SiO2) nanoparticles (NPs) as fillers in a PLA thermoplastic matrix and evaluated the strength of four 3D-printed composites at concentrations of 0.5, 1.0, 2.0, and 4.0 wt%. Compared with pure PLA, the SiO2 filler could improve the overall strength at concentrations up to 1 wt%. Gayer et al. [125] chose a selective laser sintering (SLS) process to develop a solvent-free polylactic acid/calcium carbonate composite powder. Four different grades of PLA were selected to prepare composite powders with a combination of 75 wt% of PLA and 25 wt% of calcium carbonate, and it was found that the composite with the lowest intrinsic viscosity (1.0 dL/g) showed the best processability using SLS. In bone tissue engineering, the use of carbon nanotube composites to enhance PLA contributes to cell proliferation and osteoblast differentiation [126]. Kim et al. [126] synthesized polylactic acid (PLA)–carbon nanotube (CNT) filaments using ME technology. It was demonstrated that the composites exhibited improved mechanical properties and no cytotoxicity compared to the pure polymers.

Polyethylene glycol (PEG) is a hydrophilic polymer consisting of a linear and a linear neutral polyether, and is widely used in biomedical applications such as bone tissue engineering and drug delivery. It is also an important material for surface modification [127]. PEG is known as one of the most commonly used materials in biomedical applications due to its excellent properties of biocompatibility and low toxicity, and representative materials are PEG hydrogels [128]. PEG hydrogels are often cross-linked with other polymers and used as scaffolds processed by 3D printing. For example, mixed with acrylic resins, they can be printed using stereolithography (SLA) or digital light projection (DLP). However, it is difficult to disperse energy in PEG hydrogels. Ge et al. [129] investigated solutions to this limitation. In the presence of succinic acid and mercaptosuccinic acid as dicarboxylic acids, PEG derivatives were designed via melt polycondensation of triethylene glycol (PEG (150)) and high-molecular-weight PEG. Through the self-assembly of esterified PEG (150) segments and the oxidation of sulfhydryl groups, all-PEG hydrogels with elastic nanospheres as giant cross-linkers were prepared. They exhibited excellent ductility and sub-recovery while maintaining excellent biocompatibility. In the preparation of BTE scaffolds, other polymers are often used in combination with PEG to improve the properties. Zhang et al. [130] melt-blended polylactic acid with polyethylene glycols (PEGs) of different molecular weights and found that low-molecular-weight PEGs could reduce the complex viscosity to improve processability. Wang et al. [96] synthesized a series of PEGylated PGS (PEGS) with 20% to 40% PEG content and a ratio of 0.67 to 2 carboxyl to hydroxyl groups, using a thermal curing process. They demonstrated that the PEGS elastomers around 20PEEGS-1.0C/H and 40PEGS-1.5C/H had ideal mechanical properties, degradation behavior, and cell viability, and could significantly improve the mechanical strength of calcium phosphate scaffolds. As shown in Figure 5a, the synthetic tube had a uniform wall thickness (2 mm inner diameter and 2 mm in thickness) and good toughness, proving that both 20PEGS-1.0C/H and 40PEGS-1.5C/H are ideal artificial vascular substitutes for potential vascular construction. Therefore, the optimized PEGS are promising for BTE.

Polycaprolactone (PCL), a biodegradable polyester produced by ring-opening polymerization of ε-caprolactone, is a US Food and Drug Administration (FDA)-approved biodegradable material. It is widely used in biomedical applications [131], is especially suitable for BTE, and can be printed via 3D printing methods such as FDM and SLS. Due to its hydrophobicity and crystallinity, it degrades slowly [132], and therefore synthesis with other polymers is an option for speeding up degradation and reducing costs. Davila et al. [133] fabricated PCL scaffolds and PCL composites reinforced with b-tricalcium phosphate (b-TCP) using a new AM technology called micro-screw extrusion printing. The results proved that scaffolds with a porosity of 55% and pore size of 450 μm had good mechanical properties, and the PCL composite reinforced with b-TCP had better mechanical properties and hydrophilicity.

### 4.3. Other Polymers

Polyetheretherketone (PEEK), a high-performance polymer, has many excellent properties including chemical stability, wear resistance, and good biocompatibility and elastic modulus. Due to its excellent mechanical properties and processing performance, it has become an alternative to metal implants. As a polymer, it avoids limitations such as the stress shielding present in metal implants, and therefore it can be used in bone tissue engineering as a bone implant. However, it has certain limitations such as low biological activity, and it cannot form good osseointegration with bone tissue, and therefore various studies have been carried out on improving its performance. Zhu et al. [134] introduced different contents of Ta nanoparticles (1 wt% to 9 wt%) into PEEK to improve its performance. The results for cell adhesion, collagen secretion, and osteogenesis-related gene expression were better in 3% Ta PEEK and 5% Ta PEEK. The results showed that the addition of tantalum nanoparticles improved the osseointegration ability of PEEK. Jin et al. [105] utilized graphene-modified carbon-fiber-reinforced polyetheretherketone (CFR-PEEK) and found that the microstructural parameters and average mineral deposition rate of graphene-modified CFR-PEEK implants were significantly better than those of CFR-PEEK implants (*p* < 0.05). Therefore, a coating on the surface of the PEEK implant could be considered to improve its biological activity and osseointegration. Ma et al. [135,136] prepared HA and PEEK composites and found that the osteogenic capacity of PEEK could be significantly improved using HA. J. Ma et al. [137] prepared N-HA and PEEK blended materials using 3D weaving, self-retention, and hot pressing methods. It was found that the new preparation methods could effectively reduce the negative impact of HA on the mechanical properties of PEEK, while retaining the ability of HA to improve the biological activity of PEEK. Acrylamide-based hydrogels can be used in matrix drug delivery systems. Sabbagh et al. [138] found that reducing the dosage of acrylamide from 1 g to 0.5 g could reduce the cytotoxicity of acrylamide-based hydrogels. In addition, cellulose hydrogels, as biodegradable and biocompatible materials, can also be applied in biomedicine. Their derivatives, such as HPMC and NaCMC, exhibit intelligent behaviors towards biological variables such as temperature, pH, and ionic strength, enabling hydrogels to be applied in vivo. Although cellulose is not bioabsorbable, functionalized cellulose with biodegradable extracellular matrix domains with bioactivity, i.e., hydrogels, can be used as scaffold biomaterials for bone tissue engineering [139].

In summary, polymers have been widely used due to their higher biocompatibility, degradability, and non-toxicity. Natural thermal polymers especially, alone or in combination, can be used as scaffolds, hydrogels, micro-nanospheres, etc., in bone tissue engineering. The most widely used natural polymers include silk fibroin, chitosan, and collagen, and widely used synthetic polymers include PLA, PEG, PCL, etc. At present, the AM technologies commonly used for the above polymers are FDM and SLS, and VP technology is mostly used for photopolymers. However, polymers (such as chitosan) generally have poor mechanical properties compared to metals. Therefore, they are often combined with other materials to improve the mechanical properties.

## 5. Challenges and Future Perspectives

In summary, AM technology has developed rapidly with amazing advantages since its birth, especially the advantage of being capable of personalization of the products. The products of AM processes have been used in many fields. These processes are developing rapidly in the biomedical industry and have attracted widespread attention in the field of BTE. They overcome the limitations of traditional fabrication and enable the fabrication of complex bone scaffolds. However, there are still some challenging problems.

In terms of additive manufacturing technology, the following four issues are raised, and relevant suggestions are given.

(1) Design tools should be optimized. Existing design tools have certain limitations for designers. For example, the model files for porous scaffolds are large and difficult to import into commercial AM machines. Therefore, the development of design software connected to the AM machine could be considered, to set up a more user-friendly design module. The future design tool should eliminate unnecessary information in the model file to reduce the size of the model file, for example, eliminating unimportant duplicate information, so that it can be easily imported into the AM machines.
(2) Components should be considered for structural and multidisciplinary topological optimization. In the comprehensive optimization of structural design, many factors should be considered, such as broadband vibration and material fatigue under cyclic loading. The designed components should have other properties such as thermal properties, controlled biodegradability, etc., while maintaining the necessary mechanical properties. Future work in this field should consider introducing multi-physics-driven volume design, digitally integrating multi-scale features and multi-type materials to achieve functional fusion of structures.
(3) AM processes require real-time monitoring to control the production process. The standardization of 3D printing is one of the urgent problems to be solved at present. In the next step, the corresponding 3D printing equipment certification standards should be established, with internal monitoring and closed-loop control of the production process so that the ideal prediction model and effect can be achieved.
(4) The preparation of scaffolds with complex properties is difficult. Defective bone tissue will contain cortical bone and cancellous bone, resulting in gradients of changes in mechanical properties. Therefore, the mechanical properties of integrated bone tissue scaffolds will vary greatly, and their structures will be more complex, requiring better 3D printing processes.

In terms of materials, four issues are raised and relevant suggestions are given.

(1) Material design theory should be refined. By establishing the intrinsic relationship between composition, process, and performance, a structure that meets the requirements is designed according to the properties of the materials. Future work in this field should consider combining artificial intelligence with material selection to achieve intelligent selection of materials by establishing a professional material database.
(2) Bone scaffold materials should have antibacterial or anticancer properties for some bone injuries caused by infection or bone resection due to tumors. Without antibacterial or anticancer capabilities, existing bone scaffolds will inevitably lose their effectiveness, for pathological reasons. Therefore, the development of bone scaffold materials with antibacterial or anticancer capabilities is urgently needed. In the next step in development, some antibacterial or anticancer substances could be added to existing bone scaffold materials to achieve this.
(3) The degradation rate of bone implant scaffolds should be adjusted. The internal environment of the human body is relatively complex. Although a large number of studies have been conducted to control the degradation rate of bone implant materials, there are still many factors that affect the degradation behavior, such as the microstructure. At the same time, components generated by the degradation of degradable materials must be analyzed and evaluated for safety, especially for long-term implant materials, to avoid significant negative effects on the human body.
(4) The preparation of composite materials should be improved. There has been a great deal of research on composite materials (such as multifunctional bio/synthetic composites) aimed at improving the performance of scaffolds. However, the capacity of the added material, whether the mixed material has the original biocompatibility and degradability, and the preparation method of the new composite material will all affect the performance. Therefore, the preparation of composite materials should be comprehensively considered in terms of biocompatibility, morphology, multi-layered structure, biodegradability, and growth factors that promote osteogenesis.

## 6. Conclusions

The production of bone implants involves many processes such as structural design, material selection, molding, and surface treatment. This review is mainly aimed at material selection and the methods used in forming processes, i.e., AM technology. In terms of AM technologies, in order to meet the needs of bone implants, the manufacturing method must have high efficiency and low cost. FDM, PBF, and DED are the most commonly used processes; they can obtain excellent performance and are more suitable for bone tissue engineering than other AM technologies. In terms of material selection, metal biomaterials such as titanium, tantalum, magnesium, iron, and their alloys have many problems such as stress shielding, anisotropy, degradation problems, etc., but these are still the mainstream materials for bone implants, and there have been more attempts to solve the above problems. However, since the effect of the decomposed products of metal materials is still unknown, polymers and polymer composite materials may become the main materials for bone implants in the future. At the same time, new materials such as shape memory materials are developing rapidly, and these will be a research focus in future biomedical engineering.

Although the focus of this review is on AM processes and biomaterials, the prepared scaffold structure should also be considered, as the structure also determines the performance of the scaffold. The emergence of smart materials has promoted the development of 4D printing, where materials can respond to external stimuli, providing new ideas for the printing of bone implants. Therefore, future work may also consider the application of 4D printing and smart materials in the biomedical field.

## Figures and Tables

**Table 1:** Advantages and disadvantages of additive manufacturing processes and commonly used materials in tissue engineering.

Methodology	Energy Source	Advantages	Limitations	Materials	Refs.
Material Jetting (MJ)	Thermal energy and UV light	Multi-material printing, smooth printing surface	High cost, poor mechanical properties	Photosensitive resin, thermoplastic, metal	[5]
Binder Jetting (BJ)	Thermal energy	Manufacture large and complex parts at low cost	Poor mechanical properties, requires post-processing, expensive and time-consuming	Polymer powder	[11]
Vat Photopolymerization (VP)	Laser	Fast processing speed, high surface quality, and precision of manufactured parts	High cost, high environmental requirements, complex operation	Photopolymer	[12]
Powder Bed Fusion (PBF)	Laser or beam	Design flexibility, good resolution, and low material waste	Long printing time, residual stress, need post-processing	Metal powders, polymer powder	[13,14,15]
Material Extrusion (ME)	Thermal energy	Light pollution, diverse material options, and low cost	Anisotropic, high porosity	Thermoplastic	[16,17]
Directed Energy Deposition (DED)	Laser or electron beam or plasma	Multi-material printing, cost-effective, and good mechanical properties	Limited complexity, high surface roughness, post-processing required	Metal powders, filamentary metal	[18]
Sheet Lamination (SL)	laser or beam	Low cost and fast manufacturing of large parts	Material wastage, difficult to manufacture in-house	Sheet metal, ceramics, composite fibers	[19]

**Table 2:** Advantages and disadvantages of common metals and their applications in bone tissue engineering.

Metal	Advantages	Limitations	Applications	Refs.
Titanium alloys (Ti)	Light weight, high specific strength, high corrosion resistance, good biocompatibility	Poor hardness and friction properties, possible cytotoxicity	Metallic implants such as joints and skull	[46]
Tantalum alloys (Ta)	Appropriate mechanical strength, high corrosion resistance, good biocompatibility, bone bioactivity	High cost, high density, stress shielding	Porous implants, small implant components, implant coatings	[47]
Magnesium alloys (Mg)	Suitable mechanical properties, adjustable biodegradation, density and elastic modulus are closest to those of the human body	Extremely high degradation rate results in poor tissue fixation and protection in chlorine-containing environments	Bone screw, vascular stents, implants for temporary use	[48]
Ferrous alloys (Fe)	Acceptable biocompatibility, high stretchability, strength, low cost, higher thermal conductivity	Iron degradation and release of alloying elements negatively affects cells	Short-term implants, surgical tools	[49]

**Table 3:** Characteristics of common polymers and their applications in bone tissue engineering.

Polymer		Merits	Applications	Refs.
Natural polymers	Silk fibroin(SF)	Excellent biocompatibility, degradability, tissue integration, and oxygen and water permeability	Membrane to guide bone regeneration	[100]
	Chitosan	Biocompatibility, biodegradability, non-toxic, hydrophilic	Porous bone scaffold	[101]
	Collagen	Biocompatibility, biodegradability, immunogenicity	Tissue engineering bone scaffold	[102]
Synthetic polymers	Polylactic acid(PLA)	Excellent mechanical and thermal properties, good processability, low impact on the environment	Tissue engineering, biomedical implants	[103]
	Polyethylene glycol (PEG)	Biocompatibility, water permeability, low toxicity, non-immunogenic	Drug delivery, tissue engineering, surface modification	[104]
	Polycaprolactone (PCL)	Excellent degradability, blend compatibility, mechanical properties similar to natural scaffolds, hydrophobicity, crystallinity	Long-term bone implants	[103]
Other polymers	Polyether ether ketone(PEEK)	Chemical stability, excellent heat resistance and processability, friction properties, good biocompatibility, elastic modulus close to that of human bone	Replacing metal as bone implant	[105]

## Data Availability

The data presented in this study are available on request from the corresponding author.
